# MSC-Based Cell Therapy in Neurological Diseases: A Concise Review of the Literature in Pre-Clinical and Clinical Research

**DOI:** 10.3390/biom14050538

**Published:** 2024-04-30

**Authors:** Xiaorui Zhang, Qihong Kuang, Jianguang Xu, Qing Lin, Haoming Chi, Daojin Yu

**Affiliations:** 1 University Key Laboratory for Integrated Chinese Traditional and Western Veterinary Medicine and Animal Healthcare in Fujian Province/Fujian Key Laboratory of Traditional Chinese Veterinary Medicine and Animal Health, Fujian Agriculture and Forestry University, Fuzhou 350002, China; 2 College of Life Sciences, Fujian Agriculture and Forestry University, Fuzhou 350002, China

**Keywords:** MSCs, neurological diseases, animal studies

## Abstract

Mesenchymal stem cells (MSCs) are multipotent stromal cells with the ability to self-renew and multi-directional differentiation potential. Exogenously administered MSCs can migrate to damaged tissue sites and participate in the repair of damaged tissues. A large number of pre-clinical studies and clinical trials have demonstrated that MSCs have the potential to treat the abnormalities of congenital nervous system and neurodegenerative diseases. Therefore, MSCs hold great promise in the treatment of neurological diseases. Here, we summarize and highlight current progress in the understanding of the underlying mechanisms and strategies of MSC application in neurological diseases.

## 1. Background

Mesenchymal stem cells (MSCs) exist in almost all tissues and are a type of multipotent stem cell. These cells can be extracted from a variety of tissues, including bone marrow, the umbilical cord, adipose tissue, and the placenta, and can be easily expanded [1,2]. MSCs possess the ability to self-renew and differentiate in multiple directions, offering promising prospects for addressing different tissue damages [3,4,5,6,7]. In recent two decades, many studies need references for breakthroughs in using MSCs to treat cardiovascular diseases, nervous system diseases, autoimmune diseases, and bone injuries. The etiologies of diseases related to the central nervous system are complex. Advanced understandings of the physiological and pathological characteristics of human nervous system diseases have been achieved by establishing a number of animal models, including for Alzheimer’s disease (AD), multiple sclerosis (MS), spinal cord injury (SCI), stroke, etc. [4,5,6,7]. Additionally, novel therapeutics can be evaluated on these animal models [8,9]. Recent research has shown that ongoing neurogenesis occurs from neural progenitor cells in specific areas of the central nervous system (CNS) throughout an individual’s lifespan. However, insufficient endogenous neural stem cells limit the endogenous repair of the damaged nervous system [10]. Moreover, the abnormal microenvironment can dampen the reparative process of neural stem cells when brain damage occurs [11,12]. Therefore, MSCs can be an ideal candidate in treatment of neurological diseases.

## 2. Characteristics of MSCs

MSCs possess the capability to transform into various mesenchymal cell types. The criteria set forth by the International Society for Cellular Therapy to characterize human MSCs include: (1) adhering to plastic in vitro; (2) expressing CD105, CD73, and CD90 while lacking CD45, CD34, CD14 or CD11b, CD79a or CD19, and HLA-DR on the cell surface; and (3) demonstrating the ability to differentiate into osteoblasts, adipocytes, and chondroblasts under standard culturing conditions [13]. Prior research has indicated that MSCs from varying origins possess distinct functional characteristics that could impact their efficacy in treating neurological disorders [14]. For example, MSCs derived from adipose tissue demonstrated enhanced levels of CD34, PODXL, CD36, CD49f, CD106, and CD146 surface markers, as well as greater potential for adipogenic differentiation when compared to MSCs sourced from bone marrow [15]. In addition, placenta-derived MSCs possess better immunosuppressive functions compared with umbilical cord MSCs [16]. MSCs are a promising cell source for cell therapy for various degenerative diseases, owing to their excellent properties. The discovery of the self-renewal and multi-directional differentiation of MSCs initiated their potential application in tissue repair via replacing damaged parenchymal cells. Apart from being capable of differentiating into mesoderm cells, MSCs can also undergo neural cell differentiation in specific conditions or microenvironments. Other characteristics of MSCs, including low immunogenicity properties, strong immunoregulatory effects, and abundant production of trophic factors, contribute to their therapeutic effects [17,18].

In addition, it was reported that MSCs can sense the chemokine signals released by damaged tissues and locate to the brain through the blood–brain barrier (BBB), thereby illustrating their potential in the treatment of neurological diseases [19]. Many studies have demonstrated that the therapeutic effects of MSCs on neurological diseases are related to the induction of neurogenesis, astroglial activation, the enhancement of axon growth and synaptic connections, anti-apoptosis, anti-inflammation, and a reduction in oxidative stress. A variety of bioactive molecules, immunoregulators, and growth factors produced by MSCs contribute to these characteristics [20]. Within this group of growth factors, platelet-derived growth factor (PDGF), fibroblast growth factor (FGF), and epidermal growth factor (EGF) have the ability to enhance tissue regeneration [21]. Brain-derived neurotrophic factor (BDNF), neurotrophin-3 (NT-3), and glial-derived neurotrophic factor (GDNF) can promote nerve cell survival [22,23], nerve axon regeneration, and facilitate the differentiation of endogenous neural stem cells and endogenous repair [24]. Vascular endothelial growth factor (VEGF) [21] and Ang-1 secreted by MSCs can activate endothelial cells [25], enhance blood supply, and improve metabolism, thus promoting angiogenesis and vascular repair [21]. The quality of infused MSCs could affect their therapeutic potential. Aging MSCs undergo a gradual flattening and hypertrophy in morphology, resulting in changes in their ability to proliferate, form clones, differentiate, exhibit immune characteristics, confer telomerase activity, migrate, and adhere [26]. After long-term amplification, MSC telomere shortening results in reduced self-renewal and immunomodulatory functions. However, the immunosuppressive effect of MSCs can be promoted by increasing the number of passages of umbilical cord vein MSCs due to their increased purity and major compatibility with culture conditions [27]. Similarly, the loss of osteogenic potential in elderly BM-MSCs is mediated by the *p53* gene through the miR-17 pathway [28]. Interestingly, in cardiovascular disease, it was found that miR-10a overexpression in the BM-MSCs of elderly people can activate the AKT signaling pathway and improve the angiogenesis of the ischemic mouse heart [29]. Additionally, recent studies have shown that hypoxic preconditioning significantly enhances the secretion of VEGF from aged MSCs and improves the cell viability of neurons challenged by ischemic stroke [30,31]. These investigations have been important for the development of efficient stem cell-based therapeutic approaches.

## 3. Clinical Studies of MSCs in the Treatment of Neurological Diseases

Numerous trials involving the treatment of neurological disorders with MSCs have been conducted in recent years, yet there remains a lack of sufficient efficacy data. A search of the literature on ClinicalTrials.gov, using the terms “Parkinson’s disease” and “transplantation”, yielded 16 trials (as of November 2020). Among the 16 studies, the cell source in 4 studies was MSCs. Moreover, a search on ClinicalTrials.gov resulted in the identification of 30 studies pertaining to “stroke” and “transplantation”. Approximately 50–60% of these studies involved the utilization of MSCs or similar cell types. There is a consequent need to clarify what is meant by the details of the trials being unknown. Some preliminary efficacy results revealed that MSCs can be safely and effectively used to treat neurological diseases, such as AD, MS, SCI, and stroke. For example, the injection of 3.0 × 10^6^ cells and 6.0 × 10^6^ cells into the hippocampus and thalamus of AD patients can improve neuropsychiatric symptoms, although less pathological improvement measured by PiB-PET was observed [32]. Additionally, no significant side effects appeared in AD patients during treatment. MSCs were also applied in the treatment of MS patients. The intrathecal injection of autologous BM-MSCs to PPMS and SPMS patients alleviated the symptoms and signs of MS as determined by the assessment of neurological symptoms and MRI scans. In addition, intravenous and intrathecal injection of autologous BM-MSCs into progressive patients improved neurological function, suppressed lymphocyte proliferation, and increased the proportion of immunosuppressive CD4^+^CD25^+^Tregs [33]. Moreover, BM-MSCs administrated by intravenous injection can improve the structure, physiology, and function of visual symptoms of MS patients [34]. In an initial report by Geffner et al., it was found that, in eight instances of spinal cord injury (SCI), patients received BM-MSCs through various methods, including intraspinal, intrathecal, and intravenous routes. Magnetic resonance imaging showed structural variations in the spinal cord of a few patients after the administration of BM-MSCs. The research indicated that the use of BM-MSCs through different pathways is viable, secure, and could potentially enhance the overall well-being of individuals with SCI [35]. Another clinical study showed that autologous BM-MSCs improved the clinical symptoms of 50% of patients [36]. A similar phase 1 trial of autologous BM-MSC transplantation in 18 SCI patients was performed and the results demonstrated that 7 patients underwent an improvement of one grade, while two patients saw an improvement of two grades [37]. A long-term follow-up study of intravenous autologous MSC transplantation in patients with ischemic stroke showed that no significant side effects were observed, and the follow-up MRS score was decreased compared with the control group [38]. Taken together, MSC administration can be safe in the treatment of multiple neurological diseases. However, the efficacy of MSCs should be further investigated to elucidate their indications by performing well-designed clinical studies and deciphering their therapeutic mechanisms. In the following sections, we discuss pre-clinical and clinical studies relating to the therapeutic effects of MSCs in different neurological diseases and highlight the underlying mechanisms.

## 4. Alzheimer’s Disease

Alzheimer’s disease (AD) is a significant degenerative condition affecting the central nervous system, and it is the predominant form of dementia in older individuals. AD cases are prevalent among people over 65 years of age, and they mainly show symptoms of progressive memory impairment, cognitive dysfunction, and language impairment, which seriously affect social and life functions [39]. The typical pathological markers of AD are excessive amyloid β-protein (Aβ) deposition in the brain and tau protein neurofibrillary tangles [40]. The extracellular plaques formed by the accumulation of Aβ are neurotoxic and can induce neuron loss dominated by cholinergic nerves in the cerebral cortex and hippocampus. The deposition of Aβ in the hippocampus destroys synaptic plasticity and impairs learning and memory [41]. As an age-related disease, early-onset AD (EOAD), a subtype of AD, begins before the age of 65. Changes in three distinct genes—amyloid precursor protein (APP), amyloid beta (A4), presenilin 1 (PSEN1), and presenilin 2 (PSEN2)—have been pinpointed as the primary culprits behind early-onset Alzheimer’s disease (EOAD) [42]. Late-onset Alzheimer’s disease (LOAD) typically affect individuals aged 65 years and older. Apolipoprotein E (ApoE) stands out as the primary contributor linked to heightened vulnerability to LOAD [43]. There have been many pre-clinical studies on the MSC-mediated treatment of AD. Rodents are the main experimental animals used in AD research. Park et al. showed that MSCs can induce neuronal development and neurite growth by co-culturing human-derived MSCs with neural stem cells (NSCs) derived from the subventricular zone of 5XFAD mice [44]. In addition, Lee and colleagues employed an experimentally induced model of Alzheimer’s disease by introducing amyloid beta (Aβ) into the dentate gyrus of the hippocampus of C57BL/6 mice. The intracerebral implantation of bone marrow-derived mesenchymal stem cells (BM-MSCs) into the brains of mice with induced Alzheimer’s disease resulted in enhanced cognitive function and decreased levels of Aβ compared to mice that underwent a sham transplantation. The reduction in Aβ deposits coincided with the activation of microglial cells. The morphology of the activated microglia transitioned from a branched shape to an amoeboid shape, indicating microglial phagocytosis [45]. Babaei et al. used two methods to build AD models, one being an age-induced rat AD model and the other an ibotenic acid (Ibo)-induced rat AD model. The findings from the Morris water maze (MWM) experiments indicated that treatment with BM-MSCs notably enhanced cognitive function and memory in models of age-related and ibotenic acid-induced memory deficits [46]. Yokokawa et al. transplanted 3 × 10^5^ BM-MSCs into APP/PS1 transgenic mice via the tail vein. A reduction in Aβ deposition was observed in the mouse brain following treatment with MSCs compared to the control group [47]. EPR imaging revealed a shift in the redox status of mouse brains upon MSC treatment. Kim et al. conducted a study where hUCB-MSCs were transplanted into the hippocampus of a transgenic mouse model of AD for varying durations of 10, 20, or 40 days. The results demonstrated an upregulation of neprilysin (NEP) expression in the mouse brains, along with reduced Aβ42 plaques in the hippocampus and other regions, accompanied by active migration of hUCB-MSCs towards Aβ deposits [48]. The above progress shows that hUCB-MSCs are capable of alleviating the symptoms of AD to varying degrees after transplantation, and possess good potential in the treatment of AD. In recent years, many scholars believe that combining MSCs with gene therapy may be more effective in treating AD, but further exploration is still needed. Some clinical trials on the MSC-mediated treatment of AD have been conducted, but the results are not as good as those from pre-clinical studies. Biological safety has been confirmed in clinical experiments, but the changes in AD pathophysiological processes were not significant. Animal experiments have demonstrated that hUCB-MSCs display the ability to reduce the occurrence of Aβ42 plaques in various regions including the hippocampus. This reduction appears to be linked to the movement of hUCB-MSCs towards Aβ deposits [48,49]. The variance observed between animal studies and human trials could be attributed to several factors. Initially, in the assessment of clinical treatments, PiB-PET imaging may not be as effective at identifying soluble amyloid or diffuse amyloid plaques compared to animal models [50]. Next, variations in the microenvironment of Alzheimer’s disease (AD) in humans compared to animal models might lead to varying reactions to mesenchymal stem cells (MSCs). For instance, the APP-PS1 transgenic mice utilized in earlier animal trials exhibited an accumulation of Aβ deposits without any neuronal loss or inflammation in the brain. In contrast, human specimens displayed both amyloid and tau pathologies, resulting in subsequent neuronal loss and inflammation. Additionally, previous postmortem investigations revealed that over half of clinically diagnosed AD patients have other mixed pathologies along with Aβ plaques and tau tangles [46,47]. Hence, varied pathological brain conditions could elucidate the varying reactions to MSC therapy. Another potential reason is that in mouse models of AD, xenogeneic transplants are utilized, while human clinical studies utilize autologous transplants. Consequently, considering evolutionary relationships, it is feasible that species of lower rank might experience greater advantages from MSCs derived from species of higher rank compared to the opposite scenario [48]. In order to promote the development of new clinical therapies for neurodegenerative diseases, further research is needed to evaluate the therapeutic effect of, safety of, and new cell or gene therapy methods involving MSCs from various tissue sources. We compared pre-clinical and clinical studies from four aspects: administration route, stem cell type, cell dose, and clinical index, which provides more accurate guidance for clinical research. The details are shown in Table 1.

## 5. Multiple Sclerosis

Multiple sclerosis (MS) is an autoimmune disease characterized by progressive axonal demyelination of the central nervous system. MS mainly manifests as visual impairment, paresthesia, and ataxia [51,52]. The etiology, although unclear, presumably is associated with genetic, environmental, and other factors. The neuroinflammation caused by MS usually leads to neurodegenerative lesions, axon loss, and synaptic dysfunction. During inflammation, innate immune cells (monocytes/macrophages and dendritic cells) and adaptive immune cells (T and B lymphocytes) are continuously recruited into the brain, and eventually cause autoimmune inflammation and axonal demyelination [53]. Based on clinical features, multiple sclerosis (MS) can be classified as relapsing–remitting (RRMS), secondary progressive (SPMS), primary progressive (PPMS), and progressive-relapsing (PRMS), collectively known as benign or malignant MS. The current method for diagnosing MS relies on McDonald’s Diagnostic Criteria, which integrates magnetic resonance imaging (MRI), cerebrospinal fluid (CSF) examination, and characteristic lesions detected through visual evoked potentials (VEPs). The treatment of MS includes acute relapse management, disease correction therapy (DMTS), and symptomatic treatment [54]. MS has a huge socioeconomic impact because it mainly affects young adults. Research shows that the cost of lost jobs and productivity far outweighs the cost of health and social care in the UK. Therefore, it is very necessary to develop an economical and effective treatment [55]. MSCs are considered to be the most effective method for treating patients with MS due to their ability to differentiate into other cell types [56]. Rodents are the most commonly used experimental animals. Some pre-clinical experiments have been conducted to study the MSC-mediated treatment of MS, and some results have been obtained, but there are also some undesirable points. Gordon et al. showed MSC administration by peritoneal injection reduced lesion areas and demyelination in an EAE mouse model [57]. Grigoriadis et al. administered autologous MSCs to the ventricles of EAE mice, and revealed the neuroprotective effects of MSCs [58]. Yousefi et al. injected AD-MSCs into an EAE mouse model by intraperitoneal and intravenous routes, which increased spleen Treg numbers and IL-4 levels, as well as reduced IFN-γ production and cell infiltration in the brain [59]. Another study reported that green fluorescent protein (GFP) transgenic MSCs were cultured and amplified in vitro and locally microinjected into the T11 demyelinated lesion area of MS rats. The results of an electron microscopy assay revealed that the demyelinated spinal cord could be remyelinated, and the remyelinated myelin gained the characteristics of central and peripheral nerves; in addition, their conduction speed was improved after treatment with MSCs. Remyelinated myelin also contained cells labeled with GFP, which indicates that MSCs migrated to myelin basic membrane proteins [60]. Despite these challenges, the intravenous infusion of MSCs faces difficulties in passing the lung filter and being rejected by hosts with non-compatible MHCs, which hinders their long-term engraftment in the CNS [61,62]. Nonetheless, the infusion of allogeneic MSCs has shown effectiveness in various animal models of neurological conditions [58,63]. Clinical studies have documented the significant therapeutic benefits of MSCs in treating MS. However, whether MSCs are clinically capable of protecting neural tissue and ultimately promoting neurogenesis remains unclear. Mohyeddin et al. injected 10 MS patients with autologous MSCs intrathecally. Their results showed that EDSS remained unchanged in four patients, worsened in five patients, and improved in only one patient, while MRI remained unchanged in seven patients. Thus, MSCs may not improve the clinical course and restore neural function when patients experience chronic nerve injury and secondary dysfunction. The other difference between the results obtained from pre-clinical experiments and clinical experiments is that MSCs have shown good biological safety in pre-clinical experiments, but exert adverse effects in some clinical experiments. This may be due to the improper amount of MSCs injected, including overdosing, in clinical trials [64]. The second reason may be the injection method of MSCs in clinical trials. The injection of IT is a procedure that invades the injection site, causing inflammation, edema, and trauma. Additionally, a longer treatment cycle is carried out for several months and shows significant therapeutic effects in clinical trials, but the treatment cycle of pre-clinical experiments is short. This may lead to MHC rejection caused by the implantation of MSCs in clinical experiments. In order to provide more accurate guidance for clinical research, we compared pre-clinical and clinical studies from four aspects: administration route, stem cell type, cell dose, and clinical index. The details are shown in Table 2.

## 6. Spinal Cord Injury

Spinal Cord Injury (SCI) is a serious global public health problem that imposes a significant financial and emotional burden on patients and their families [67]. SCI patients have sensory and motor dysfunction below the normal level, and their chronic complications include respiratory, cardiovascular, urogenital, and gastrointestinal dysfunction, as well as increased systemic motor nerve spasm [68]. The pathological mechanism of SCI is very complicated. Local injury areas are full of perivascular, endothelial, bone marrow, and meningeal cells, resulting in inflammation, fibrosis, and vascular rupture. The inflammatory response further results in enzyme activation, mediator release, inflammatory cell migration, glial activation, and neuronal tissue degradation [69,70]. Further, astrocytes separate necrotic tissue from healthy tissue, but “glial scars” are formed due to excessive proliferation [71], which affects axonal regeneration, extension, and fusion [72]. SCI is divided into primary and secondary injuries. In primary SCI, cells appear disintegrated, necrosis and apoptosis occur in the damaged area because of the action of physical tension, and vascular rupture, tissue edema, and other manifestations are also present. During the period of secondary injury, a large number of macrophages, T cells, microglia cells, and neutrophils infiltrate the blood–spinal cord barrier, and result in the release of various inflammatory factors, and cause a series of inflammatory damages [73]. Many pre-clinical studies have demonstrated that transplantation of MSCs is an effective method to alleviate and treat SCI. Pre-clinical subjects selected have included not only rodents (46.7%), but also dogs (33.3%), monkeys (13.3%) and pigs (6.7%) [74]. Rodent models of SCI are mainly used in pre-clinical studies of SCI and are the basis for demonstrating the efficacy and safety of transplanted MSCs in the treatment of SCI. Yutaka Nishio et al. reported that hUCB-MSC transplantation improved functional recovery and promoted the regeneration or sparing of axons in SCI [75]. Sung-Rae Cho and colleagues illustrated that spinal cord injury (SCI) rats experienced a modest enhancement in locomotor rating scale at 10 weeks following the infusion of human umbilical cord blood-derived mesenchymal stem cells (hUCB-MSCs). Additionally, hUCB-MSCs’ intervention in SCI rats also indicated the presence of somatosensory-evoked potentials [76]. Another study showed that BM-MSCs, transplanted with a lumbar puncture method, migrated to the site of injury and significantly improved locomotor and sensory behavior score in a dose dependent manner [77]. In their study, Ramil Hakim and colleagues examined the cellular reaction of MSCs after being transplanted into SCI-affected animals. The findings indicated that MSCs acquired characteristics resembling immune cells, including elevated levels of CD45 and MHC-II expression, as well as increased activity in genes associated with cytokine synthesis, phagocytosis/endocytosis, and the immune response [78]. Other animal models, such as dogs and pigs, can more accurately reflect the efficacy and safety of MSC transplantation in SCI. Hak-hyun RYU et al. compared the effects of AD-MSCs, BM-MSCs, and UCB-MSCs on axon regeneration in SCI dogs, indicating that UCB-MSCs are more effective in promoting nerve regeneration, nerve protection, and reducing the inflammatory response [79]. In addition, previous research has demonstrated that autologous BM-MSC transplantation succeeded in a pig model of chronic paraplegia [80]. Additionally, the transplantation of mesenchymal stem cells (MSCs) has the potential to stimulate the growth of new neurons and improve neurological function in spinal cord injury (SCI) models of non-human primates [81,82]. The efficacy of MSC therapy has been demonstrated in various animal models of SCI. Nevertheless, additional investigations involving primate models are required prior to clinical implementation. Clarifying pre-clinical research is the premise and important guarantee of clinical research. Numerous pre-clinical trials have demonstrated that transplanting MSCs into the site of injury can be an efficacious treatment for spinal cord injuries. In contrast to the varied use of MSCs in pre-clinical investigations, clinical studies currently focus on transplanting autologous BM-MSCs from the patient to the injured area of the spinal cord. The main reason may be that autologous BM-MSCs are easy to obtain, with low costs of culturation and a high survival rate following transplantation. More SCI-related clinical trials are being prepared and recruited for. These experiments included a variety of MSCs, such as hUCB-MSCs and AD-MSCs [83]. In order to provide more accurate guidance for clinical research, we compared pre-clinical and clinical studies from four aspects: administration route, stem cell type, cell dose, and clinical index. There was no significant difference in the type or number of MSCs colonized, as the number of cells was not uniform. Bruna et al. indicated that using MSCs at a density of 8 × 10^5^ cells produced optimal outcomes, implying that this quantity was a suitable option for treating SCI [84]. The details are shown in Table 3.

## 7. Stroke

Stroke is the second leading cause of death in the world and increasingly becoming a global burden [86]. Stroke is more common in cerebrovascular diseases, and generally refers to cerebral ischemia, which is also the cause of disability and death. The pathogenesis of stroke is influenced by multiple factors. Brain physiology and blood flow are two vital factors in stroke. When the local blood flow of the brain is interrupted, the energy and oxygen supply required by the brain neurons are interrupted, resulting in the absence of an ATP-dependent intracellular ion concentration gradient and enhanced apoptosis of brain neurons. In ischemic stroke, the Notch1, NF-κB, p53, HIF-1α, and Pin1 signaling pathways, which control the fate of neurons, are activated. Depending on the extent of ischemia, the results can be different. A moderate level of ischemia results in the upregulation of pro-survival genes. However, serious ischemic hypoxia induces the expression of genes associated with the death of neurons [87,88]. Additionally, the release of extracellular excitatory amino acids (EAAs) is positively correlated with the vulnerability to cerebral ischemia. Specifically, the accumulation of glutamate greatly increases the probability of lesions [89]. In recent years, the MSC-mediated treatment of stroke has been studied in a variety of animal models. The animal models used in ischemic stroke research include rodents (89.2%), rabbits (1%), dogs (3%), and monkeys (1%). Among them, the rat stroke model (88.1%) is most widely used [90]. The animal model of transient middle cerebral artery occlusion (TMCAO) involves inducing ischemic-associated neurodegeneration in rodents, which is the main pre-clinical model of stroke and can be used to evaluate the efficacy and safety of transplanted MSCs in the treatment of stroke [91]. Some scholars have proved that the nasal administration of MSCs can reduce white matter injury after TMCAO and enhance the somatosensory function of newborn rats after stroke [92]. In addition, MSC transplantation significantly enhanced the mRNA expression of BDNF and nerve growth factor (NGF) in ischemic stroke rat models [93]. MSC transplantation also reduced ischemia-induced neuronal apoptosis and infarct size by downregulating p-JNK, thus improving neurological function and behavior scores in ischemic stroke model rats [94]. Moreover, MSC therapy has also showed potential in improving neural function in other animal models of stroke. For example, in a dog model of thromboembolic cerebral ischemia, hUCB-MSCs increased the production of BDNF and VEGF, reduced infarct area, and restored damaged nerve function [95]. Similarly, transplantation of hBM-MSCs in the ischemic tissues of cynomolgus monkeys enhanced the expression of interleukin-10 (IL-10), decreased neuronal apoptosis, increased proliferation of cells in the subventricular zone (SVZ), and improved neurological function [96]. Although most current studies on stem cell transplantation have confirmed the effective therapeutic effects of MSCs for stroke, there is a certain gap between different kinds of animal modeling and human clinical treatment. Therefore, new experiments and practices are needed to verify its long-term safety and effectiveness. MSCs are the main reservoir of stem cells employed in most pre-clinical and clinical investigations related to stroke. With the increasing number of clinical trials, the therapeutic effects of MSC transplantation will be shown. Nonetheless, variations exist between pre-clinical and clinical research endeavors. First, pre-clinical trials are mainly based on rodent models of cerebral ischemia compared with clinical trials, which cannot fully reproduce the mechanism of ischemic stroke in humans, especially the response of the immune system. Second, most pre-clinical trials use MSCs from healthy subjects, mainly as the ability of MSCs from stroke patients to secrete bioactive molecules is not yet recognized. However, some studies have proved little difference between the two [97], so using allogeneic stem cells may be a good option in clinical application. Finally, there are some risks in clinical neurosurgery due to the inconsistency of monitoring and evaluation levels between pre-clinical and clinical studies. Therefore, ethical, technical, and medical issues must be overcome in translating pre-clinical studies into clinical application. In order to provide more accurate guidance for clinical research, we compared pre-clinical and clinical studies from four aspects: administration route, stem cell type, cell dose, and clinical index. The details are shown in Table 4.

## 8. Underlying Mechanisms of MSC-Mediated Treatment of Neurological Diseases

### 8.1. Migration

The foundation of utilizing MSCs for treating neurological disorders lies in the capacity of these cells to travel to the area of tissue injury. In actuality, the existence of the blood–brain barrier (BBB) restricts the passage of macromolecules and cells, which makes the treatment of neurological diseases very difficult. Numerous research studies have demonstrated that MSCs have the ability to traverse the BBB via areas of compromise [100]. There is a diverse range of cytokine and chemokine receptors expressed by MSCs, enabling them to promptly adapt to varying microenvironmental conditions [101]. In particular, MSCs possess CXCR4, which serves as the receptor for SDF1α (CXCL12), a chemoattractant that is produced at sites of inflammation, facilitating the precise localization of MSCs to inflammatory sites [102]. Exogenous MSCs can home to most inflammation sites in different diseases, such as inflammatory bowel disease [103], cardiac infarction [104], and tumors [105,106]. It is worth noting that the ability of MSCs to migrate to injured sites is affected by many factors [107]. Firstly, MSCs secrete paracrine growth factors, including vascular endothelial growth factor, insulin-like growth factor, hepatocyte growth factor, and fibroblast growth factor, which play a significant role in guiding MSCs to the site of injury [107]. Payne et al. demonstrated that the expression of chemokine receptors on MSCs is regulated by inflammatory cytokines such as IFN-γ [107]. Additionally, the culture environment of MSCs appears to be a critical factor in the manifestation of homing proteins [108]. In particular, prolonged culturing of cells leads to a reduction in the levels of chemokine receptors and the chemotactic capabilities of MSCs. Furthermore, the source of MSCs plays a significant role in their ability to migrate. According to Rossi et al., Ad-MSCs and UC-MSCs exhibit higher expression of migratory molecules compared to BM MSCs [109].

### 8.2. Immunomodulation

Considering that MSCs are able to directly or indirectly, via paracrine interactions, interact with immune cells, MSCs are reported as immunomodulator cells [110]. When inflammation caused by neurological diseases occurs, MSCs are mobilized to the damaged site and respond to inflammatory cytokines, therefore exerting immunoregulatory effects through the release of various mediators [111]. Certain autoimmune diseases like multiple sclerosis (MS) typically manifest following the migration and infiltration of pathogenic T cells into the central nervous system (CNS), leading to the secretion of various pro-inflammatory cytokines, including IL-1β, IL-6, IL-17, tumor necrosis factor (TNF)-α, and interferon (INF)-γ [112]. The combination of cytokines TNF-α, IL-1α, and IL-1β has been shown to induce MSCs to express high levels of multiple chemokines and inducible nitric oxide synthase (iNOS) [110]. The proximity of T cells to MSCs through the synergistic action of chemokines can be suppressed by high concentrations of secreted nitric oxide (NO) [110]. In addition, MSCs were reported to be able to regulate the ratio of Th2/Th1 [111]. For example, BM-MSC transplantation in EAE mice obviously increased Th2 cytokine production and significantly reduced inflammatory infiltration and demyelination [113]. Corcione et al. revealed that MSCs can arrest the cell cycle of B cells and suppress their division and antibody production [114]. MSCs can also attenuate MS via suppressing the proportion of CD5^+^ IL-10^+^ B cells [66]. Additionally, a previous study displayed that MSCs can exert immunosuppressive activity via the regulation of regulatory T-cell (Treg) function [111]. Kim et al. demonstrated that intrathecal injections of BM-MSCs into amyotrophic lateral sclerosis (ALS) patients elevated the ratio of Tregs, thus protecting neuronal cells [115]. The immunosuppressive effect of MSCs is usually mediated by several soluble factors derived from MSCs, including TGF-β1, prostaglandin E2 (PGE2), hepatocyte growth factor (HGF), indoleamine-pyrrole 2,3-Dioxygenase (IDO), NO, and IL-10 [65]. It is worth noting that MSCs from different sources have different types of secreted factors. For example, compared with UC-MSCs and Ad-MSCs, the soluble factors secreted by BM-MSCs had a stronger regulatory effect on T cell responses [107]. Another point worth noting is that the immunosuppressive activity of MSCs in neurological diseases depends on the direct contact between MSCs and T cells to a great extent [111]. Numerous studies have demonstrated that, during inflammatory responses in neurological disorders, certain adhesion molecules like CD274, vascular cell adhesion molecule-1, and galectin-1 are upregulated in mesenchymal stem cells (MSCs). This upregulation leads to an increase in cell–cell contact and a reinforcement of immunomodulatory functions [116,117]. In addition, previous studies showed that insufficient expression of intercellular cell adhesion molecule-1 (ICAM-1) or vascular cell adhesion molecule 1 (VCAM-1) weakened the immunosuppressive effects of MSCs [117].

### 8.3. Differentiation and Neuroregeneration

At present, the most difficult challenge in treating neurological diseases is the inability of neural tissue to fully repair itself [118]. Mesenchymal stem cells (MSCs) can undergo distinction into various mesenchymal lineages, such as osteoblasts, adipocytes, and chondroblasts, in both in in vitro and in vivo settings [119]. In a study by Kopen GC et al., it was demonstrated that MSCs, when introduced into the central nervous systems of young mice, have the ability to take on the morphological and phenotypic traits of astrocytes and neurons [120]. The important discovery of Kopen GC et al. provides reliable evidence that MSCs can differentiate into nerve cells to repair the nervous system damage caused by neurological diseases. A study revealed that administering MSCs through the nasal passage to patients with neurodegenerative disorders resulted in rapid migration of the stem cells within 1 to 2 h after transplantation. However, very few MSCs were found in CNS lesions after 72 h [121]. Despite this, a noticeable improvement in symptoms related to the disease was noted. The apparent recovery was not attributed to the MSCs transforming into nerve cells but rather to the release of neurotrophic and neurotropic factors through exosomes or microvesicles (MVs). It was reported that MSCs reduce inflammation and repair tissue damage via secreting soluble factors, neurotropic factors, and microvesicles [100]. Moreover, transplanted hMSCs promoted the survival of endogenous cells, including injured neurons, immature oligodendrocytes, and oligodendrocyte progenitor cells in vitro via secreting factors [122]. Previous research has shown that the environment in which MSCs are located has an important impact on the types of neurotrophic factors they produce [123]. For example, Munoz et al. revealed that intraperitoneal injection of human amnion-MSCs in EAE mice could inhibit programming cell death and improve neuronal function via secreting NGF, ciliary neurotrophic factor (CNTF), and BDNF [124]. In addition, HGF was reported to be the main soluble factor secreted by MSCs [56]. HGF plays an important role in EAE recovery, whereas blocking of HGF and its tyrosine kinase receptor inhibited the neurotrophic activity of MSCs [56].

### 8.4. Promoting Axon Regeneration

Research has shown that the transplantation of BM-MSCs leads to the survival and migration of 2′,3′-Cyclic nucleotide 3′-phosphodiesterase (CNP) positive cells and Schwann cells to the site of injury, providing support for axon regeneration and remyelination following SCI [125]. Okuda et al. illustrated that an ascorbic acid-induced BM-MSCs sheet also aids in the treatment of SCI through autologous transplantation without the need for a scaffold. Their research indicated that the spinal cord defect was filled with a folded BM-MSCs sheet, which facilitated the regeneration of axons (Bone marrow stromal cell sheets may enhance axonal regeneration and functional recovery while inhibiting glial scar formation following spinal cord injury in rats) [74]. Furthermore, biomaterial-supported MSCs transplantation can fulfill multiple functions for treatment of SCI. The specific three-dimensional microarchitectures control the release of bioactive molecules and improves the survival and retention of transplanted cells at the lesion site. At the same time, it serves as a physical substrate for cell adhesion and is suitable for cell seeding and axonal linear growth [85]. All these results indicated that MSCs could inhibit the glial scar and provide a suitable habitat for injured axon to regeneration.

### 8.5. MSCs–Derived Exosome Releasing

Exosomes, known as extracellular vesicles, can transmit biological data across significant distances, impacting both typical and irregular cellular and tissue functions [126]. In recent years, there has been much evidence showing the great potential of MSC-derived exosomes in treating neurological diseases [127,128,129]. Katsuda et al. revealed that exosomes derived from adipose tissue-derived MSCs displayed neprilysin-related enzyme activity and were involved in reducing β-amyloid levels in neuroblastoma cells [130]. Confirmation indicated that exosomes from murine MSCs derived from adipose tissue enhanced the viability of human neuroblastoma cells and safeguarded hippocampal neurons in mice against oxidative harm [129]. In addition, exosomes obtained from murine adipose tissue-derived MSCs were also proved to enhance remyelination and promote the activation of oligodendroglial progenitors [129], which was also confirmed by Bonafede et al. [131]. The above results highlight the potential of MSC-derived exosomes to be employed as a therapeutic tool for treating neurological diseases. Existing studies have shown that exosomes obtained from BM-MSCs and adipose-derived MSCs have good therapeutic effects on neurological diseases [98,99,129,131,132]. However, no study has been performed to evaluate the neuroprotective potential of exosomes obtained from other types of MSCs. Additional investigation is required to characterize the proteins, mRNAs, and miRNAs in exosomes isolated from MSCs sourced from different origins. Furthermore, a comparative analysis of the therapeutic properties of exosomes derived from diverse MSCs is essential.

## 9. Conclusions and Future Prospects

Mesenchymal stem cells have a wide range of potential applications in the treatment of neurological disorders. Notably, MSC-based treatments have not lived up to expectations, although a large number of pre-clinical and clinical trials have been explored. For example, the clinical application of autologous MSCs is limited by their time-consuming preparation. Variabilities in cell sources, doses and dosing intervals, isolation, culture, and expansion protocols present problems that have yet to be resolved. The homing ability, differentiation efficiency, and regeneration rate of MSCs should be optimized to improve the efficacy of treatment. It is necessary to further explore the critical genes that determine the differentiation of MSCs into neural cells, illustrate the cellular and molecular mechanisms underneath the differentiation, and find ways to regulate cell migration and integration. Through further research in vivo and in vitro to determine the molecular interaction between the transplanted cells and the host, we can establish the best transplantation method to ensure long-term biological safety after transplantation. These methods can be further optimized by studying the regeneration mechanism of neural tissue, and finally applied to clinical research from a reliable perspective. MSCs transplanted through veins will encounter first-pass metabolism in the liver and pulmonary circulation metabolism, and a high concentration of cells may lead to pulmonary vein embolism; thus, how to choose the appropriate dose remains to be demonstrated. Although arteriovenous and subarachnoid space transplantation has opened up a reliable path for cell transplantation, the high demand for cells is a major barrier which includes a high economic cost of production, processing, and storage before the cells can be made available to patients. In addition, it is necessary to consider whether MSCs need to be induced or modified before transplantation to improve the targeting, transfection efficiency, continuous expression, and controllability of MSC gene therapy. There are many methods for making a nervous system animal model, but different modeling methods actually reflect different pathological mechanisms of the same disease. In addition, the evaluation indicators selected in the production of animal models are various, and the non-specificity of the indicators will also lead to differences in disease phenotypes and drug effects. More crucially, there are many distinctions in the biological characteristics of animals and humans, and the results of pre-clinical animal experiments need to be verified in clinical experiments. Therefore, we need to further explore the principle of disease pathogenesis and build appropriate animal disease models that mimic human disease based on the degree of similarity between different species and humans in terms of neural functional structure, immunity, and metabolism. Although there are still many unknown fields for MSCs at present, with an improved understanding of MSC biology and revolutionized technology, we believe that MSC transplantation is likely to bring breakthroughs for the treatment of neurological diseases in the near future.

## Figures and Tables

**Table 1:** Differences between pre-clinical and clinical studies of MSCs in AD.

	Pre-Clinical Study	Clinical Study	Reference
Administration route	1. Transplantation into the hippocampus; 2. Tail vein injection.	Brain	[45,47,50]
Cell type	1. hUCB-MSCs; 2. BM-MSCs.	hUCB-MSCs	[39,44,45]
Cell dose	3 × 10^5^ cells transplanted into the patient	1. Low dose (3.0 × 10^6^ cells); 2. High dose (6.0 × 10^6^ cells).	[32,45,47]
Clinical index	1. Levels of Aβ deposition; 2. Levels of Secretase (BACE1); 3. Tau hyperphosphorylation; 4. Behavior test (MWM); 5. Redox status of mouse brains evaluated by EPR imaging; 6. Synaptic regeneration evaluated by level of synapsin I.	1. Levels of Aβ deposition; 2. Levels of Secretase (BACE1); 3. Tau hyperphosphorylation.	[4,41,43,46,47,48,50]

**Table 2:** Differences between pre-clinical and clinical studies of MSCs in MS.

	Pre-Clinical Study	Clinical Study	Reference
Administration route	1. Intravenous; 2. Intrathecal; 3. Intraperitoneal.	1. Intravenous; 2. Intrathecal.	[33,34,58,59,61]
Stem cell type	MSCs from bone marrow, fat, fetal, and dental tissues	BM-MSCs	[4,54,63,65]
Cell dose	Not unified		[33,58,59,63]
Clinical index	1. Immunological assessments (Th1, Th17 cells, and Th2 cells, anti-inflammatory cytokines, and IL-4); 2. Demyelination; 3. Neurological functions.	1. Immunological assessments (lymphocyte proliferation, proportion of CD4, CD25, and Treg cells, expression of FOXP3, IFN-ɤ, and TGF-β, MSC dose (1 × 10^6^–100 × 10^6^ cell), IL-4, IL-10, and IL-6); 2. Neurological; 3. MRI assessments; 4. Visual acuity; 5. Visual evoked response latency; 6. Optic nerve area.	[5,33,34,53,56,57,58,59,60,61,62,63,66]

**Table 3:** Differences between pre-clinical and clinical studies.

	Pre-Clinical Study	Clinical Study	Reference
Administration route	1. Intrathecal; 2. Intralesional; 3. Intravenous.	1. Intrathecal; 2. Intraspinal; 3. Intralesional; 4. Perilesional; 5. Intravenous; 6. Intraarterial.	[35,37,70,75,77,78,79,84]
Stem cell type	1. BM-MSCs; 2. AD-MSCS; 3. hUCB-MSCs.	BM-MSCs	[35,36,74,75,76]
Cell dose	Not unified		[77,78,80,81,84]
Clinical index	1. ASIA, AIS, SEP, MRI, EMG, Frankel score, Residual urinary volume, Barthel; 2. Behavior test (grid walk, plantar test, inclined plane, gait analysis, spontaneous motor activity, Tarlov behavior assessment); 3. Sensory test (limb pinch test, tail pinch test); 4. Cell surface protein expression (CD34, CD44, CD45, CD73, CD90, CD105, fibronectin, vimentin, laminin cellspositivity etc.); 5. Cell level test (cells were evaluated by flow cytometry, immunohistochemistry, immunocytochemistry, proliferation assay differentiation assay, confocal microscopy, and automatic cell quantification); 6. Other tests (BBB locomotor scale, electrical conduction).	1. ASIA; 2. AIS; 3. SEP; 4. MRI; 5. EMG; 6. Frankel score; 7.Esidual urinary volume; 8.Barthel.	[36,67,68,69,70,71,72,74,75,76,77,78,79,80,81,83,85]

**Table 4:** Differences between pre-clinical and clinical studies.

	Pre-Clinical Study	Clinical Study	Reference
Administration route	1. Perilesional; 2. Intravenous; 3. Intraarterial; 4. Intralesional.	1. Intravenous; 2. Intraarterial; 3. Stereotactic injection; 4. Subarachnoid injection.	[23,38,90,92,93,94,97,98]
Stem cell type	1. BM-MSCs; 2. UBC-MSCs; 3. DP-MSCs.	BM-MSCs	[30,38,90,92,94,95]
Cell dose	Not unified	MSC dose (1 × 10^8^ cell)	[23,38,90,92,95]
Clinical index	1. Behavior test (MWM); 2. Redox status of mouse brains evaluated by EPR imaging; 3. Immunological assessments (proportion of CD40, CD80, and CD86 cells, anti-inflammatory cytokines, TNF-α, IL-1β, IL-6, and IL-4); 4. Neurological functions.	1.Neurological functions; 2. MRI assessments; 3. NIH stroke scale (NIHSS); 4. Barthel index (BI); 5. Fugl–Meyer assessment (FMA); 6. Functional independence measure (FIM).	[23,30,31,38,87,88,90,91,92,93,94,95,96,97,98,99]

## Abbreviations

MSCs: mesenchymal stem cells, AD: Alzheimer’s disease, MS: multiple sclerosis, SCI: spinal cord injury, PDGF: platelet-derived growth factor, FGF: fibroblast growth factor, EGF: epidermal growth factor, BDNF: brain-derived neurotrophic factor, NT-3: neurotrophin-3, GDNF: glial-derived neurotrophic factor, VEGF: vascular endothelial growth factor, EOAD: early-onset AD, A4: amyloid beta, APP: amyloid precursor protein, LOAD: late-onset AD, ApoE: apolipoprotein E, NGF: nerve growth factor, IL-10: interleukin-10, NSCs: neural stem cells, MWM: Morris water maze, GFP: green fluorescent protein, CNP: 2′,3′-cyclic nucleotide 3′-phosphodiesterase, EAAs: excitatory amino acids; SVZ: subventricular zone, MV: microvesicles, CNTF: ciliary neurotrophic factor, VCAM-1: vascular cell adhesion molecule 1, ICAM-1: intercellular cell adhesion molecule-1, PGE2: prostaglandin E2 (PGE2), HGF: hepatocyte growth factor, IDO: indoleamine-pyrrole 2,3-Dioxygenase, ALS: amyotrophic lateral sclerosis, TNF: tumor necrosis factor, INF: interferon, Treg: regulatory T-cell, PDGF-AB: platelet derived growth factor-AB, IGF-1: insulin growth factor-1.
